# Efficacy of vitamin C supplementation during pregnancy in the prevention of preterm birth: a systematic review and meta-analysis

**DOI:** 10.61622/rbgo/2025rbgo1

**Published:** 2025-03-17

**Authors:** Ana Gabriela Alves Pereira, Gabriela Oliveira Gonçalves Molino, Ana Clara Felix de Farias Santos, Maírla Marina Ferreira Dias, Nicole dos Santos Pimenta, Pedro Henrique Costa Matos da Silva

**Affiliations:** 1 Universidade Estadual Paulista "Júlio de Mesquita Filho" São Paulo SP Brazil Universidade Estadual Paulista "Júlio de Mesquita Filho", São Paulo, SP, Brazil.; 2 Universidade Federal de Ciências da Saúde de Porto Alegre Porto Alegre RS Brazil Universidade Federal de Ciências da Saúde de Porto Alegre, Porto Alegre, RS, Brazil.; 3 Universidade Cidade de São Paulo São Paulo SP Brazil Universidade Cidade de São Paulo, São Paulo, SP, Brazil.; 4 Universidade Federal de Campina Grande Campina Grande PB Brazil Universidade Federal de Campina Grande, Campina Grande, PB, Brazil.; 5 Universidade Federal do Estado do Rio de Janeiro Rio de Janeiro RJ Brazil Universidade Federal do Estado do Rio de Janeiro, Rio de Janeiro, RJ, Brazil.; 6 Universidade Federal de Goiás Goiânia GO Brazil Universidade Federal de Goiás, Goiânia, GO, Brazil.

**Keywords:** Vitamin C supplementation, Vitamin E, Pregnancy, Premature birth, Gestational age, Fetal membranes, premature rupture, Ascorbic acid, Intensive care units, neonatal

## Abstract

**Objective:**

Preterm birth is a leading global cause of neonatal mortality and morbidity, with oxidative stress playing a role in its pathogenesis. Vitamin C, a powerful antioxidant, may help reduce this risk. This study assessed the effectiveness of vitamin C supplementation, both alone and with vitamin E, in preventing preterm birth compared to a placebo.

**Data source:**

Databases were systematically searched in PubMed, Cochrane and Embase in December 2023 and updated in May 2024.

**Study Selection:**

Included RCTs evaluated vitamin C's effect on preterm birth and related neonatal outcomes.

**Data collect:**

Statistical analyses used a random-effects model for pooled risk ratios (RR) and 95% confidence intervals (CI). Heterogeneity was assessed with the I² statistic.

**Data synthesis:**

Seventeen RCTs (21,567 patients) were analyzed. Vitamin C supplementation showed no significant difference compared to placebo for preterm birth (RR 1.04; 95% CI 0.96, 1.14). No significant differences were observed for neonatal death (RR 0.77; 95% CI 0.55, 1.08), NICU admission (RR 1.03; 95% CI 0.95, 1.13), preterm PROM (RR 1.04; 95% CI 0.63, 1.71), or birth weight (MD 52.41; 95% CI −19.65, 124.47). A slight decrease in gestational age was observed (MD 0.26; 95% CI −0.02, 0.55).

**Conclusion:**

Vitamin C supplementation alone or in combination with vitamin E does not significantly prevent preterm birth or improve related neonatal outcomes.

## Introduction

Preterm birth, defined as any birth occurring before 37 weeks of gestation, affects approximately 15 million babies annually, representing a high global rate of approximately 11%.^(1)^ It stands as the leading cause of neonatal and childhood mortality, accounting for 70-80% of neonatal deaths and 80% of associated complications in premature infants.^(2)^ Survivors of preterm birth often face long-term health challenges, including cerebral palsy, sensory impairments, developmental delays, behavioral issues, and an increased risk of chronic diseases later in life.^(3)^

The majority of preterm births are spontaneous, and research indicates that oxidative stress plays a significant role in their pathogenesis. Oxidative damage to fetal membranes disrupts the balance between antioxidants and pro-oxidants, leading to complications such as fetal growth restriction and other pregnancy-related issues.^(4)^ Vitamin C, or ascorbic acid, is a water-soluble antioxidant crucial for collagen metabolism and the integrity of chorioamniotic membranes. Unlike most animals, humans cannot synthesize vitamin C endogenously and must obtain it through dietary intake. During pregnancy, the demand for vitamin C increases as it is actively transported across the placenta, resulting in a decline in maternal plasma vitamin C levels.^(5)^

Inadequate levels of ascorbic acid during pregnancy have been proposed as a risk factor for premature rupture of the chorioamniotic membranes (PROM) and subsequent spontaneous preterm birth. Evidence suggests that vitamin C supplementation can mitigate oxidative stress and endothelial dysfunction, potentially extending the latency period before birth.^(6)^

Previous meta-analyses have examined a variety of studies, including both randomized controlled trials (RCTs) and observational studies, up to March 2015. However, more recent and larger RCTs have been conducted with more rigorous methodologies.^(7)^ These systematic review and meta-analysis aim to evaluate the efficacy of vitamin C supplementation, alone or in combination with vitamin E, compared to placebo in preventing preterm birth. The objective is to determine whether vitamin C supplementation during pregnancy provides tangible benefits in reducing the incidence of preterm birth and improving neonatal outcomes.

Therefore, this analysis seeks to provide a comprehensive assessment of the potential role of vitamin C in mitigating the risks associated with preterm birth, thereby contributing to the body of knowledge that informs clinical practice and public health policies.

## Methods

These systematic review and meta-analysis was performed and reported in accordance with the Cochrane Collaboration Handbook for Systematic Review of Interventions and the Preferred Reporting Items for Systematic Reviews and Meta-Analysis (PRISMA) Statement guidelines.^(8,9)^

### Eligibility criteria

Studies were included if they met the following eligibility criteria: (i) RCT; (ii) pregnant women; (iii) vitamin C supplementation; (iv) reporting at least one outcome of interest. Exclusions were applied based on the following criteria: overlapping populations, defined as studies with overlapping institutions and recruitment periods; non-randomized studies; abstracts; studies with medically indicated preterm birth; studies involving multivitamin supplementation; and studies not published in English.

### Search strategy and data extraction

A comprehensive literature search was conducted across PubMed, Embase and Cochrane Central register of Controlled trials databases for studies meeting the eligibility criteria. The initial search was conducted in December 2023, and the search was updated in May 2024. The search strategy included the terms "vitamin C supplementation", "pregnancy" and "preterm birth", along with their synonyms. Additionally, we analyzed the references of systematic reviews and included studies to identify any other potentially eligible studies. Information regarding the definition of standard care from the randomized controlled trials (RCTs) was gathered for analysis. The outcome data underwent cross-verification, consolidation, and were then entered into the meta-analysis software.

### Endpoints and subgroup analysis

The primary outcomes included preterm birth, birth weight, gestational age (GA) at birth, neonatal intensive care unit (NICU) admission, neonatal death and preterm premature rupture of membranes (PPROM). We performed a subgroup analyses between vitamin C supplementation alone and vitamin C in combination with vitamin E supplementation. The outcomes of preterm birth, birth weight and gestational age at birth were assessed in both analysis. Additionally, we conducted a leave-one-out analysis to evaluate the impact of influential studies on the combined outcomes. This method involved systematically excluding data from individual studies and re-evaluating the remaining dataset to ensure the consistency of the pooled treatment effect. We identified study dominance whenever excluding a study altered the significance of the pooled effect size p-values, either from significant to non-significant or vice versa.^(9)^

### Quality assessment and publication bias

The quality of individual RCTs was assessed using the Cochrane Collaboration tool for assessing risk of bias in randomized trials (RoB 2).^(10)^ Two independent investigators conducted the quality assessment and any disagreements were resolved by a third author.. Each trial was evaluated across five domains: randomization process; deviations from the intended interventions; missing outcomes; measurement of the outcome; and selection of reported results. The overall design and visualization of the risk of bias assessment were crafted by Robvis.^(11)^ In order to assess potential publication bias, funnel plots were visually inspected and control lines were analysed.

### Statistical analysis

The treatment effects for binary endpoints were compared using risk ratio (RR), with 95% confidence intervals (CIs). Heterogeneity was assessed with the Cochrane Q-test and I^2^ statistics; P values > 0.10 and I^2^ values > 25% were considered to indicate significance for heterogeneity.^(12)^ We employed Mantel-Haenszel and DerSimonian and Laird random-effects models were used in all outcomes exhibiting either low or significant heterogeneity. Random effects meta-analyses were conducted to calculate pooled effect sizes, based on the following assumptions: the included studies used slightly different intervention combinations and varied patient inclusion and exclusion criteria, potentially indicating differences in care. Therefore, the variation in effect estimates could be due to within-study sampling error, between-study heterogeneity, or both.^(13)^ Data handling adhered to the guidelines provided by the Cochrane Handbook for Systematic Reviews of Interventions.^(9)^ Cochrane Review Manager Software (RevMan 5.4) was used to perform statistical analysis (Nordic Cochrane Centre, The Cochrane Collaboration, Copenhagen, Denmark). Meta-analyses were performed using the R software environment, version 4.3.0 (R Foundation for Statistical Computing).

## Results

### Study selection and baseline characteristics

**Figure 1:**
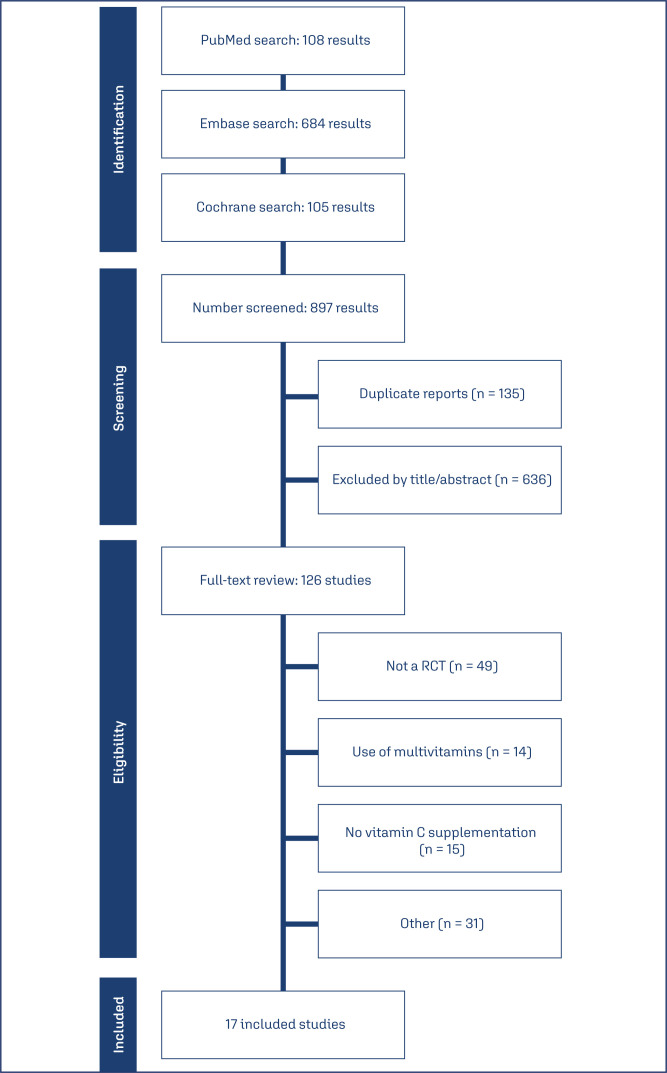
PRISMA flow diagram of study screening and selection

As illustrated in figure 1, 897 studies were identified. After removal of duplicate records and ineligible studies, 126 remained and were fully reviewed based on inclusion criteria. Seventeen manuscripts met all inclusion criteria and were included in quantitative analyses. The initial characteristics of participants in each study were largely similar across groups. The analysis included a distinct population of 21,567 patients, with 10,792 (50%) of them receiving vitamin C supplements. The mean age ranged from 22 to 31 years, and the mean BMI ranged from 20 to 30.4 kg/m^2^. Smoking was reported in 2 to 1593 patients, and 14 to 1170 women were multiparous. The mean gestational age (GA) ranged from 13 to 39.2 weeks (Chart 1 S1). Study characteristics are presented in chart 1.

**Chart 1 S1:** Search strategy

PubMed	("ascorbic acid"[Mesh] OR "vitamin C" OR "vitamin C supplementation" OR "ascorbic acid" OR "ascorbate") AND ("premature birth"[Mesh] OR "preterm delivery" OR "preterm birth" OR "fetal Membranes, premature rupture"[Mesh] OR "premature rupture" OR "preterm rupture" OR PROM OR PPROM OR "preterm prelabour rupture of membranes")	108*
Cochrane	(vitamin C OR vitamin C supplementation OR ascorbic acid OR ascorbate) AND (preterm delivery OR preterm birth OR premature rupture OR preterm rupture OR PROM OR PPROM OR preterm prelabour rupture of membranes)	105*
Embase	("vitamin C" OR "vitamin C supplementation" OR "ascorbic acid" OR "ascorbate") AND ("preterm delivery" OR "preterm birth" OR "premature rupture" OR "preterm rupture" OR PROM OR PPROM OR "preterm prelabour rupture of membranes")	684*
*Search strategy performed	in 03 December 2023 and updated in 10 May 2024	

**Chart 1:** Baseline characteristics of included studies

Study	Intervention	N° of patients I / C	Age† § (y) I / C	BMI† (kg/m^2^) I / C	Smoking (n)	GA† * (wk) I / C	Multiparous I / C
Abdulhussain et al. (2022)^(14)^	100 mg vit C	55 / 45	25.6/24.2	25.9/25.16	NA	NA	NA
Abramovici et al. (2015)^(15)^	1000 mg vit C + 400 IU vit E	788 / 763	22.4/22.2	26.2/26.6^c^	total smoking	13.1/13.1	234/260
Borna et al. (2005)^(16)^	500 mg vit C + 400 IU vit E	30 / 30	26.4/27.4	NA	9	13/14^d^,^e^	17/14
Casanueva et al. (2005)^(17)^	100 mg vit C	52 / 57	27.5/27.4	24.2/23.8^c^	NA	20/20^b^	NA
Ghomian et al. (2013)^(18)^	100 mg vit C	85 / 85	29.8/29	21.4/20.8	NA	31/31.4^a^	NA
Gungorduk et al. (2014)^(19)^	1000 mg vit C + 400 IU vit E	126 / 123	26.7/26.4	30/30.4	40	30.1/30.5	59/62
Kiondo et al. (2014)^(20)^	1000 mg vit C	466 / 466	NA	20-29^f^	2	19.3/19.7	331/337
McCance et al. (2010)^(21)^	1000 mg vit C + 400 IU vit E	379 / 382	29.5/29.6	27.6/27.4	149	14.3/14.2	189/195
Mercer et al. (2010)^(22)^	1000 mg vit C + 400 IU vit E	36 / 37	23.6/23.4	NA	NA	39.2/38.3	NA
Poston et al. (2006)^(23)^	1000 mg vit C + 400 IU vit E	1199 / 1205	31/30.9	30.3/30.3	290	18.6/18.5	599/592
Roberts et al. (2010)^(24)^	1000 mg vit C + 400 IU vit E	5087 / 5065	23.5/23.5	25.4/25.4^c^	1593	13.4/13.4	1161/1170
Spinnato et al. (2007)^(25)^	1000 mg vit C + 400 IU vit E	371 / 368	28.9/29.7	28.5/28.8^c^	102	15.6/15.7	334/334
Spinnato et al. (2008)^(26)^	1000 mg vit C + 400 IU vit E	371 / 368	28.9/29.7	28.5/28.8^c^	102	15.6/15.7	333/335
Steyn et al. (2003)^(27)^	250 mg vit C	100 / 100	28/28	24.2/24.2	86	13/14	100/100
Torky et al. (2021)^(28)^	1000mg vit C	450/455	28/27	26.3/27	NA	NA	NA
Xu et al. (2010)^(29)^	1000 mg vit C + 400 IU vit E	1167 / 1196	28.6/28.6	34.4/35.3	164	15.1/15.2	233/239
Zamani et al. (2013)^(30)^	500 mg vit C	30 / 30	24.9/24.6	NA	NA	18/18	19/22

BMI: body mass index; C, control; GA, gestational age in weeks; I, intervention; NA: not available; Vit C: vitamin C; Vit E: vitamin E.

† Mean or median;

§ Maternal age;

* At randomization

a GA at PPROM in previous pregnancy;

b GA at prenatal care;

c Prepregnancy Body mass index;

d Data regarding patients with 26-28 weeks of gestation;

e GA at the time of PROM in current pregnancy;

f Data derived from the age range of half of the patients in each group;

### Pooled analysis of all studies

**Figure 2:**
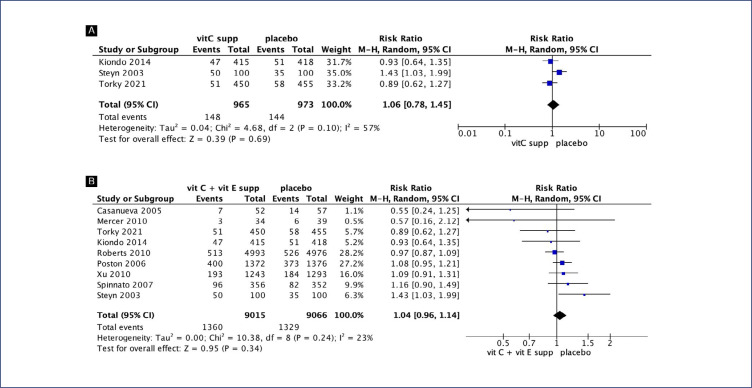
Forest plot of studies examining Preterm delivery. A: Vitamin C alone supplementation; B: Vitamin C and vitamin E supplementation

**Figure S1:**
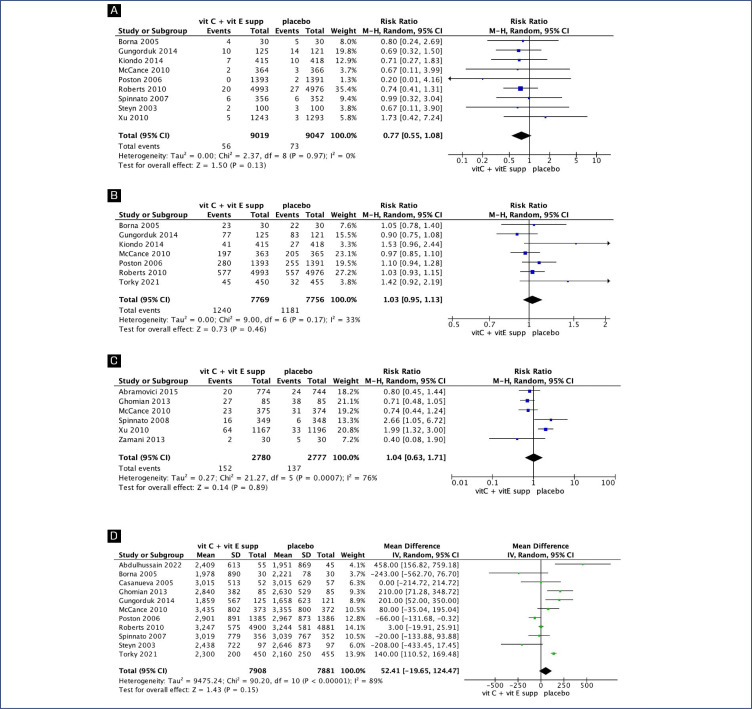
Forest plot of Vitamin C and Vitamin E supplementation

**Figure S2:**
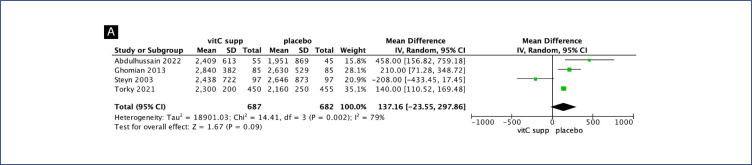
Forest plot of Vitamin C alone supplementation

**Figure 3:**
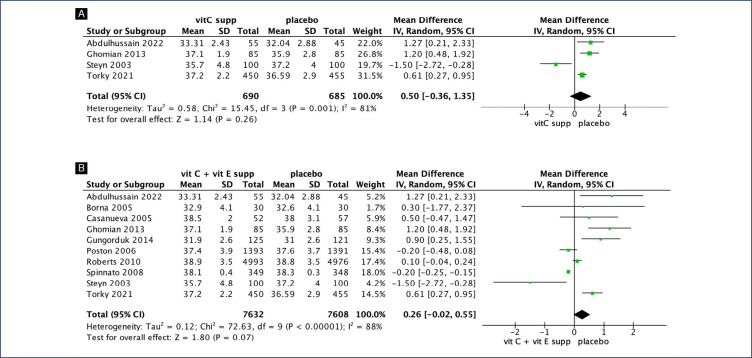
Forest plot of studies examining Gestational Age at birth. A: Vitamin C alone supplementation; B: Vitamin C and vitamin E supplementation

No clear differences were seen between pregnant women supplemented with vitamin C alone compared with placebo for preterm birth (RR 1.06; 95% CI 0.78, 1.45) (Figure 2A), gestational age at birth (MD 0.55; 95% CI −0.36, 1.35) (Figure 3A) and birth weight (MD 137.16; 95% CI −23.55, 297.86) (Figure S1). Similarly, no differences were seen between pregnant women supplemented with vitamin C in combination with vitamin E compared with placebo for preterm birth (RR 1.04; 95% CI 0.96, 1.14) (Figure 2B), neonatal death (RR 0.77; 95% CI 0.55, 1.08), neonatal intensive care unit (NICU) admission (RR 1.03; 95% CI 0.95, 1.13), preterm premature rupture of membranes (PPROM) (RR 1.04; 95% CI 0.63, 1.71) and birth weight (MD 52.41; 95% CI −19.65, 124.47) (Figure S2). However, pregnant women supplemented with vitamin C in combination with vitamin E had a small decrease in gestational age at birth (MD 0.26; 95% CI −0.02, 0.55) (Figure 3B).

A: Neonatal death; B: Neonatal Intensive Care Unit admission; C: Preterm Premature Rupture of Membranes; D: Birth Weight

A: Birth Weight

### Sub analysis in selected populations

In the sub analysis performed between RCTs with vitamin C supplementation alone and RCTs with vitamin C in combination with vitamin E supplementation, no significant differences were observed in preterm birth, birth weight and gestational age at birth between the vitamin C alone supplementation and the vitamin C in combination with vitamin E supplementation groups.

### Sensitivity analysis

**Figure S3:**
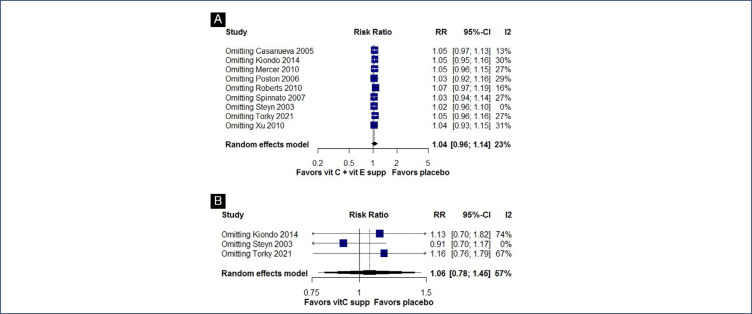
Leave-one-out sensitivity analysis of studies examining Preterm birth

We performed a leave-one-out sensitivity analysis for preterm birth in vitamin C alone and vitamin C in combination with vitamin E subgroups. This analysis is shown in figure S3. The variance among studies concerning preterm birth notably decreased when excluding Steyn et al.^(27)^ from both the vitamin C supplementation alone and vitamin C combined with vitamin E supplementation groups. Specifically, the reduction was from I^2^ = 57% to I^2^ = 0% and from I^2^ = 23% to I^2^ = 0%, respectively. This decrease likely stemmed from the distinct patient demographics in Steyn et al.,^(27)^ which differed notably from those in other trials due to a higher proportion of women with prior mid-trimester abortion or previous preterm labor. Conversely, no substantial alterations in outcome heterogeneity were evident upon the exclusion of individual studies in the leave-one-out analyses.

A: Vitamin C and Vitamin E supplementation; B: Vitamin C alone supplementation

**Figure S4:**
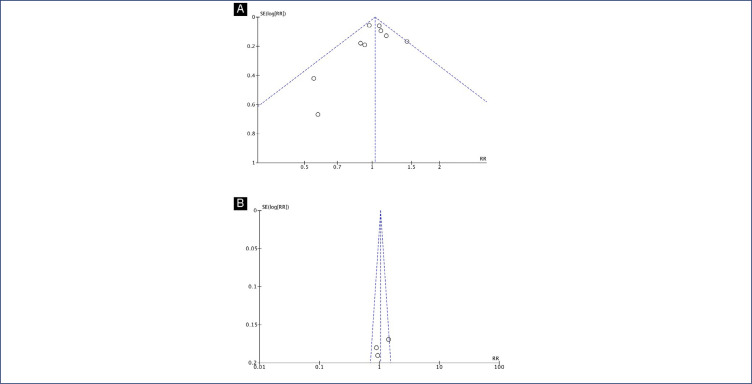
Funnel plot of studies examining Preterm birth

A: Patients supplemented with Vitamin C and Vitamin E; B: Patients supplemented with Vitamin C alone

### Risk of bias within studies and publication bias

**Figure 4:**
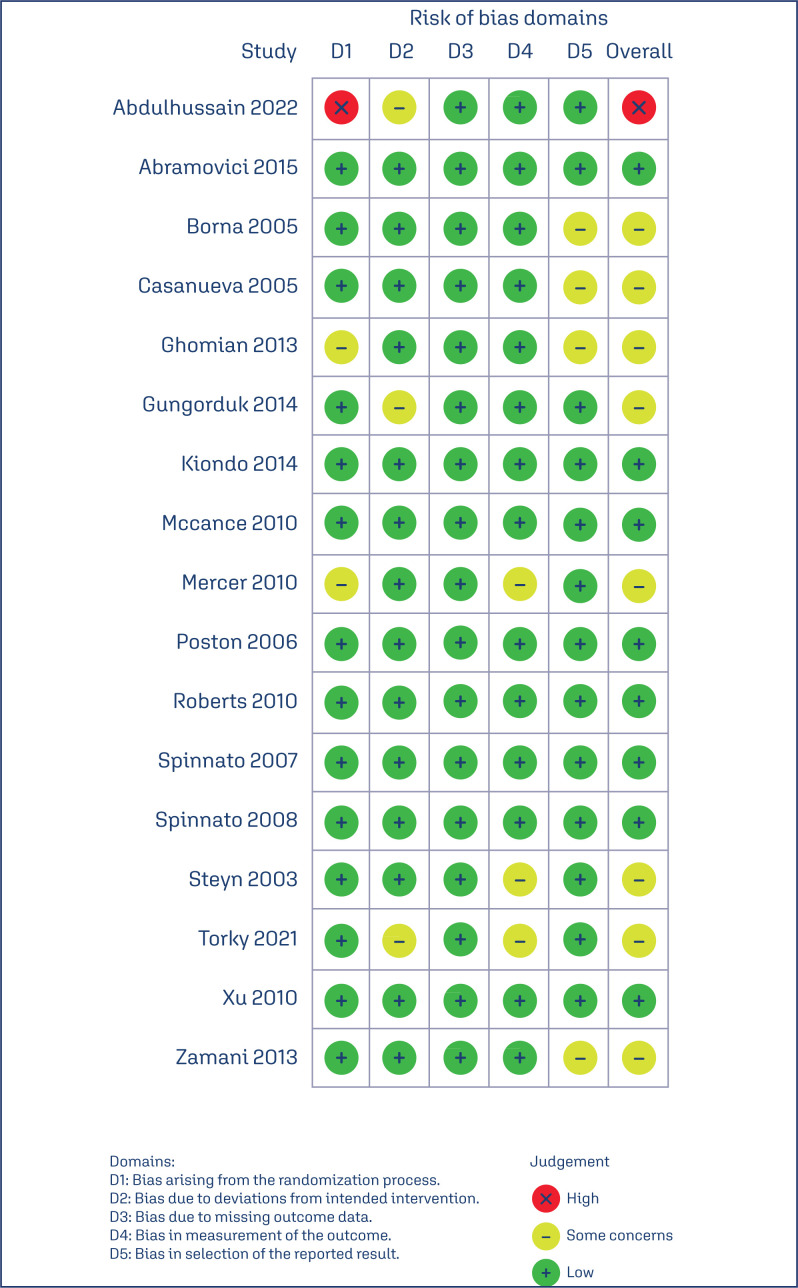
Critical appraisal of randomized controlled trials according to the Cochrane Collaboration tool for assessing risk of bias in randomized trials

The risk of individual within-study bias is represented in the RoB 2 traffic-light diagram (Figure 4). Every study was prone to performance bias because it was not feasible to blind patients and investigators during the trials. One RCT was assigned as high risk of bias due to bias arising from the randomization process and deviations to intended intervention.^(16)^ Eight RCTs raised some concerns about bias in at least one RoB 2 assessment tool domain.^(27,16–19,22,28,30)^ Of the thirteen remaining studies, all were assigned at low risk of bias.^(15, 20,21,23–26,29)^

## Discussion

In these systematic review and meta-analysis including seventeen studies and 21,567 patients, vitamin C and vitamin E supplementation was compared with vitamin C alone supplementation. The results of this review do not support routine vitamin C supplementation given that there were no beneficial effect in the supplementation with vitamin C in combination with vitamin E or vitamin C alone compared with placebo for preventing preterm birth and other neonatal outcomes, such as neonatal death, neonatal intensive care unit admission, birth weight, preterm premature rupture of membranes and gestational age at birth.

Currently, the intervention effectiveness in preventing preterm birth remains controversial. Within the ongoing review, three studies emerged favoring placebo over vitamin C alone supplementation concerning the outcome of gestational age at birth. This contrasting finding prompts questioning whether vitamin C supplementation during pregnancy could indeed be beneficial for maternal and neonatal outcomes or if it poses potential harm to the health of these individuals. The biochemical evidence based on the antioxidants properties of vitamin C suggests that the ascorbic acid plays an important role in the integrity of chorioamniotic membranes, reducing oxidative stress and endothelial dysfunction and leading to an increase in the latency period, which prevents premature rupture of the chorioamniotic membranes (PROM) and, consequently, prevents also spontaneous preterm birth.

Accordingly, there is no convincing evidence in the present literature to admit that vitamin C supplementation can be harmful during pregnancy. Further investigation is needed in this context.

It is essential to interpret our study findings while considering its limitations. To begin with, there was noticeable variability, ranging from moderate to high, in some outcomes examined, like preterm birth. Nonetheless, leave-one-out sensitivity analyses were performed, and the results remained stable after systematically excluding each study. Secondly, the differences between the studied populations must be understood, as women underwent supplementation at different gestational ages, with varying clinical profiles, histories and risk factors for preterm birth development.

A meta-analysis by Swaney et al.^(31)^ pointed out that the lack of data in the published articles on the background of vitamin C intake can be a limitation for the study of vitamin C supplementation during pregnancy. This consideration can be applied in the current review, given that some studies gathered data on vitamin C consumption through food surveys and other methods, which can be unreliable and many studies did not provide any data at all. This absence of information prevents conclusions about whether a specific intake level of vitamin C would benefit patients with poor nutritional statuses. While some studies focused on populations where poor nutrition was more relevant, the lack of specific data made it impossible to compare results from different nutritional backgrounds. Furthermore, although most studies excluded participants based on their intake of vitamin C supplements, the exclusion criteria varied across studies. Therefore, as previously mentioned, determining the confounding amount of vitamin C intake remains challenging.

Another concern regarding the analysis of vitamin C supplementation during pregnancy is that most studies include women with previous PPROM or with risk factors for developing preeclampsia, which means that vitamin C supplementation is usually evaluated in high risk population. Nevertheless, high risk pregnancies correspond to only 12% on average of pregnancies worldwide.^(32)^ This means that more research has to be made evaluating vitamin C supplementation in the general population, so that eventual side effects, harms and benefits of this intake can be measured without confounders.

## Conclusion

The data of this meta-analysis shows no clear difference between vitamin C supplementation alone or in combination with vitamin E for the prevention of preterm birth and other neonatal outcomes. These findings indicate that vitamin C supplementation during pregnancy provides no benefit or potential harms. Therefore, further research is required to elucidate the possible role of vitamin C in maternal and neonatal health.

## References

[1] Walani SR (2020). Global burden of preterm birth. Int J Gynaecol Obstet.

[2] Vogel JP, Chawanpaiboon S, Moller AB, Watananirun K, Bonet M, Lumbiganon P (2018). The global epidemiology of preterm birth. Best Pract Res Clin Obstet Gynaecol.

[3] Goldenberg RL, Culhane JF, Iams JD, Romero R (2008). Epidemiology and causes of preterm birth. Lancet.

[4] Robles R, Palomino N, Robles A (2001). Oxidative stress in the neonate. Early Hum Dev.

[5] Mousa A, Naqash A, Lim S (2019). Macronutrient and micronutrient intake during pregnancy: an overview of recent evidence. Nutrients.

[6] Prins JR, Schoots MH, Wessels JI, Campmans-Kuijpers MJ, Navis GJ, van Goor H (2022). The influence of the dietary exposome on oxidative stress in pregnancy complications. Mol Aspects Med.

[7] Rumbold A, Ota E, Nagata C, Shahrook S, Crowther CA (2015). Vitamin C supplementation in pregnancy. Cochrane Database Syst Rev.

[8] Page MJ, McKenzie JE, Bossuyt PM, Boutron I, Hoffmann TC, Mulrow CD (2021). The PRISMA 2020 statement: an updated guideline for reporting systematic reviews. BMJ.

[9] Deeks JJ, Higgins JP, Altman DG, Higgins JP, Thomas J, Chandler J, Cumpston M, Li T, Page MJ (2019). Cochrane handbook for systematic reviews of interventions.

[10] Sterne JA, Savović J, Page MJ, Elbers RG, Blencowe NS, Boutron I (2019). RoB 2: a revised tool for assessing risk of bias in randomised trials. BMJ.

[11] McGuinness LA, Higgins JP (2021). Risk-of-bias VISualization (robvis): an R package and Shiny web app for visualizing risk-of-bias assessments. Res Synth Methods.

[12] Higgins JP, Thompson SG, Deeks JJ, Altman DG (2003). Measuring inconsistency in meta-analyses. BMJ.

[13] Schandelmaier S, Briel M, Varadhan R, Schmid CH, Devasenapathy N, Hayward RA (2020). Development of the Instrument to assess the Credibility of Effect Modification Analyses (ICEMAN) in randomized controlled trials and meta-analyses. CMAJ.

[14] Abdulhussain AS (2022). The efficacy and safety of vitamin C administration to women with history of premature preterm rupture of membrane in prevention of such event in current pregnancy: randomized controlled clinical trial. J Popul Ther Clin Pharmacol.

[15] Abramovici A, Gandley RE, Clifton RG, Leveno KJ, Myatt L, Wapner RJ (2015). Prenatal vitamin C and E supplementation in smokers is associated with reduced placental abruption and preterm birth: a secondary analysis. BJOG.

[16] Borna S, Borna H, Daneshbodie B (2005). Vitamins C and E in the latency period in women with preterm premature rupture of membranes. Int J Gynaecol Obstet.

[17] Casanueva E, Ripoll C, Tolentino M, Morales RM, Pfeffer F, Vilchis P (2005). Vitamin C supplementation to prevent premature rupture of the chorioamniotic membranes: a randomized trial. Am J Clin Nutr.

[18] Ghomian N, Hafizi L, Takhti Z (2013). The role of vitamin C in prevention of preterm premature rupture of membranes. Iran Red Crescent Med J.

[19] Gungorduk K, Asicioglu O, Gungorduk OC, Yildirim G, Besimoğlu B, Ark C (2014). Does vitamin C and vitamin E supplementation prolong the latency period before delivery following the preterm premature rupture of membranes? A randomized controlled study. Am J Perinatol.

[20] Kiondo P, Wamuyu-Maina G, Wandabwa J, Bimenya GS, Tumwesigye NM, Okong P (2014). The effects of vitamin C supplementation on pre-eclampsia in Mulago Hospital, Kampala, Uganda: a randomized placebo controlled clinical trial. BMC Pregnancy Childbirth.

[21] McCance DR, Holmes VA, Maresh MJ, Patterson CC, Walker JD, Pearson DW (2010). Vitamins C and E for prevention of pre-eclampsia in women with type 1 diabetes (DAPIT): a randomised placebo-controlled trial. Lancet.

[22] Mercer BM, Abdelrahim A, Moore RM, Novak J, Kumar D, Mansour JM (2010). The impact of vitamin C supplementation in pregnancy and in vitro upon fetal membrane strength and remodeling. Reprod Sci.

[23] Poston L, Briley AL, Seed PT, Kelly FJ, Shennan AH, Vitamins in Pre-eclampsia (VIP) Trial Consortium (2006). Vitamin C and vitamin E in pregnant women at risk for pre-eclampsia (VIP trial): randomised placebo-controlled trial. Lancet.

[24] Roberts JM, Myatt L, Spong CY, Thom EA, Hauth JC, Leveno KJ (2010). Vitamins C and E to prevent complications of pregnancy-associated hypertension. N Engl J Med.

[25] Spinnato JA, Freire S, Pinto e Silva JL, Rudge MV, Martins-Costa S, Koch MA (2007). Antioxidant therapy to prevent preeclampsia: a randomized controlled trial. Obstet Gynecol.

[26] Spinnato JA, Freire S, Pinto e Silva JL, Rudge MV, Martins-Costa S, Koch MA (2008). Antioxidant supplementation and premature rupture of the membranes: a planned secondary analysis. Am J Obstet Gynecol.

[27] Steyn PS, Odendaal HJ, Schoeman J, Stander C, Fanie N, Grové D (2003). A randomised, double-blind placebo-controlled trial of ascorbic acid supplementation for the prevention of preterm labour. J Obstet Gynaecol.

[28] Torky HA, Abo-Louz A, Deif O, Moussa A, Aly R, Shata A (2021). Role of Vitamin C in prevention of preeclampsia in high-risk cases: randomized controlled trial. Int J Childbirth.

[29] Xu H, Perez-Cuevas R, Xiong X, Reyes H, Roy C, Julien P (2010). An international trial of antioxidants in the prevention of preeclampsia (INTAPP). Am J Obstet Gynecol.

[30] Zamani M, Goodarzi MT, Lavasani NS, Khosravi A (2013). Effects of ascorbic acid on serum level of unconjugated estriol and its relationship with preterm premature rupture of membrane: a double-blind randomized controlled clinical trial. Iran J Med Sci.

[31] Swaney P, Thorp J, Allen I (2014). Vitamin C supplementation in pregnancy--does it decrease rates of preterm birth? A systematic review. Am J Perinatol.

[32] Coco L, Giannone TT, Zarbo G (2014). Management of high-risk pregnancy. Minerva Ginecol.

